# Isolation, Identification, and Antimicrobial Susceptibility of *Exiguobacterium mexicanum* from a Giraffe

**DOI:** 10.3390/vetsci12100969

**Published:** 2025-10-10

**Authors:** Fei Gao, Qunchao Liang, Rui Zong, Yuqing Xie, Chenxu Zhao, Yurong Yang, Linyang Yu, Dongliang Li, Hong Duan, Wenjuan Du, Yongtao Li

**Affiliations:** 1 College of Veterinary Medicine, Henan Agricultural University, Zhengzhou 450046, China; 18736529435@163.com (F.G.); liangqc03@126.com (Q.L.); 17658403228@163.com (R.Z.); xieyuqing2023@163.com (Y.X.); zhaochenxu0818@163.com (C.Z.); yangyu7712@sina.com (Y.Y.); linyangyuhenau@163.com (L.Y.); 497222038@sina.com (D.L.); duanhong0924@126.com (H.D.); 2 International Joint Research Center of National Animal Immunology, College of Veterinary Medicine, Henan Agricultural University, Zhengzhou 450046, China; 3 Ministry of Education Key Laboratory for Animal Pathogens and Biosafety, Henan Agricultural University, Zhengzhou 450046, China; 4 Longhu Laboratory, Zhengzhou 450046, China

**Keywords:** giraffe, *E. mexicanum*, isolation, identification, antimicrobial susceptibility

## Abstract

**Simple Summary:**

*Exiguobacterium mexicanum* (*E. mexicanum*) is a facultative anaerobic, Gram-positive bacterium within the genus *Exiguobacterium*. Although it is commonly found in diverse environments—including soil, water, and extreme habitats such as hot springs and permafrost—this species is rarely linked to infections in humans or animals. This study reports the isolation and characterization of a potentially pathogenic strain of *E. mexicanum* from a giraffe, along with its antimicrobial susceptibility profile, to inform clinical treatment strategies. The pathogenicity of *Exiguobacterium* species in wildlife remains poorly understood, with very few documented infections and limited data on host range, virulence factors, and resistance patterns. This gap underscores the significance of the current isolate, suggesting that *E. mexicanum* may represent an emerging pathogen in giraffes. Notably, despite the well-documented threats to giraffe populations, there have been no previous reports of *E. mexicanum* infection in this species, highlighting the novelty of our findings and emphasizing the need for further research into its potential role as a pathogen.

**Abstract:**

In May 2025, a female giraffe in poor body condition died unexpectedly at a zoo in Henan Province, China. A bacterial strain, designated HN-1, was isolated from the heart, liver, spleen, lungs, and kidneys of the deceased animal. After 24 h of incubation at 37 °C on Luria–Bertani (LB) agar, the colonies appeared round, smooth, pale yellow, translucent, and raised. Gram staining revealed that the isolate was a Gram-positive, rod-shaped, and non-spore-forming bacterium. Based on 16S *rRNA* gene sequencing, the strain showed more than 99.7% homology with reference sequences of *E. mexicanum* from various sources in GenBank. The results of the susceptibility test showed that *E. mexicanum* was susceptible to levofloxacin, clindamycin, chloramphenicol, trimethoprim, rifampicin, tetracycline, minocycline, gentamicin, erythromycin, and doxycycline, but resistant to oxacillin, penicillin, ciprofloxacin, and linezolid. These findings provide valuable insights for the diagnosis and treatment of infections caused by *E. mexicanum* in giraffes.

## 1. Introduction

Giraffes (*Giraffa camelopardalis*), the world’s tallest terrestrial animals and largest ruminants [1], face a variety of health challenges in both wild and captive environments stemming from variations in diet [2], environmental conditions, climate [3], and susceptibility to infectious diseases. Captive giraffes are particularly vulnerable to bacterial infections [4,5,6], as exemplified by cases of *Mycobacterium avium* subsp. *paratuberculosis* identified in wild ruminants at a Mexican zoo [7], *Fusobacterium necrophorum* causing severe bilateral necrotic lesions near the tongue base and oral commissures in giraffes at Ordos Zoo [8], and *Mycobacterium bovis* associated with tuberculosis in giraffes at Greater Kruger National Park [9]. Given the dramatic decline in giraffe populations in recent decades, effective conservation strategies are crucial for ensuring their long-term survival [10].

*E*. *mexicanum* is a metabolically versatile bacterium exhibiting noteworthy biological characteristics and potential applications [11,12]. As a member of the *Exiguobacterium* genus, known for its ability to thrive across broad temperature and pH ranges [9], *E*. *mexicanum* was initially isolated and identified as one of two Gram-positive strains from brine shrimp (*Artemia*) cysts [13]. Subsequent isolations include strain A-EM from Atlantic deep-sea hydrothermal vents [14] and strain HA2 from Ilam Mountain in Iran, which demonstrates growth at 0–25 °C and tolerance up to 5% NaCl [15]. The genus has also been identified in the hyperarid Atacama Desert of Chile [16] and petroleum-contaminated permafrost [17], highlighting *E*. *mexicanum*’s adaptability to extreme environmental conditions. While direct evidence of *E*. *mexicanum* pathogenicity in animals or humans remains limited, other *Exiguobacterium* species are recognized as opportunistic pathogens with the potential to infect immunocompromised individuals [18]. Reported cases have linked the genus to community-acquired pneumonia and bacteremia, including a 2017 report of *Exiguobacterium* sp. isolated from both the blood and bronchoalveolar lavage fluid of a pneumonia patient [11]. Although the specific pathogenic potential of *E*. *mexicanum* requires further elucidation, the established pathogenicity of related species warrants continued investigation into its potential health implications.

## 2. Materials and Methods

### 2.1. Case Reports and Pathological Examination

In May 2025, a 605 kg female giraffe (ID: 41) in poor body condition died unexpectedly at a zoo in Zhengzhou, Henan Province, China. On the evening of 18 May (after 19:00), the giraffe showed reduced standing time, frequent recumbency, obvious weakness during ambulation, and an increased respiratory rate. It retained its appetite, consuming the provided foliage, but refused to drink water. On 19 May, the giraffe attempted to stand twice, each attempt lasting approximately two minutes, presenting with an unsteady gait, deep and rapid respiration, lethargy, dull eyes, and excretion of dark brown, tea-colored urine. At 11:35, it exhibited limb paddling and neck weakness, and unfortunately died despite emergency treatment. A comprehensive necropsy was subsequently performed, including a thorough pathological examination of all organs. Fresh tissue samples were sent to the Veterinary Diagnostic Laboratory of Henan Agricultural University for further analysis. Samples were collected from visceral organs (heart, liver, spleen, lungs, and kidneys) using sterile scalpels, scissors, and inoculating loops for subsequent histopathological and laboratory studies.

### 2.2. Bacterial Isolation and Morphological Observation

Under sterile conditions in a biosafety cabinet, samples from the heart, liver, spleen, and kidney were inoculated onto LB agar and blood agar plates (Biocell, Zhengzhou, China) using a sterile inoculating loop. The plates were then incubated at 37 °C for 16–24 h to facilitate colony observation. Single colonies exhibiting uniform morphology were selected and repeatedly streaked to ensure pure cultures displaying consistent colony size and morphology. These purified single colonies were subsequently inoculated into LB liquid medium for culture expansion and preserved on agar plates at 4 °C. Gram staining was performed on the purified bacterial strains, followed by microscopic examination to confirm cell morphology and Gram reaction.

### 2.3. Molecular Identification

Genomic DNA was extracted from pure bacterial colonies using a DNA purification kit (Tiangen Biotech, Beijing, China), with the extracted DNA stored at −20 °C until use as PCR templates. Molecular identification was performed through sequencing and phylogenetic analysis of the 16S *rRNA* and *recA* genes. The 16S*rRNA* gene was amplified using universal primers 27F (5′-AGAGTTTGATCCTGGCTCAG-3′) and 1492R (5′-GGCTACCTTGTTACGACTT-3′) [19]. The *recA* gene was amplified with primers recA-F (TATCGACTTCTGCCGTCTTGAAC) and recA-R(GTCATCGAAGTGTACGGACCTG).

PCR reactions were performed in a 25 µL volume, comprising 12.5 µL Premix TaqTM, 1 µL each of the forward and reverse primers, 2 µL of DNA template, and 8.5 µL of sterile ddH_2_O. The amplification protocol consisted of an initial denaturation step at 95 °C for 5 min, followed by 30 cycles of denaturation at 95 °C for 30 s, annealing at 59 °C for 30 s, and extension at 72 °C for 20 s, with a final extension at 72 °C for 10 min. PCR products were verified by agarose gel electrophoresis and subsequently submitted to Shangya Biotechnology Co., Ltd. (Henan, China) for sequencing. The resulting sequences were compared to sequences in GenBank using the BLAST algorithm (National Center for Biotechnology Information, NCBI, https://blast.ncbi.nlm.nih.gov/Blast.cgi, accessed on 20 August 2025). Phylogenetic trees, based on both 16S *rRNA* and *recA* gene sequences, were constructed using the neighbor-joining method implemented in MEGA 7.0 software, with bootstrap analysis performed using 1000 replicates.

### 2.4. Antibiotic Susceptibility Testing

A bacterial suspension adjusted to a 0.5 McFarland standard (100 μL) was evenly spread over Mueller-Hinton agar plates (HOPEBIO, Qingdao, China) using a sterile spreader. Antimicrobial susceptibility test disks (OXOID) were placed on the agar surface with sterile forceps, with three replicate plates per disk type. After allowing the plates to stand for 3 min, they were inverted and incubated at 37 °C for 24 h. The diameter of each inhibition zone was measured using a vernier caliper, and results were expressed as the mean value. Since the Clinical and Laboratory Standards Institute (CLSI) has not established interpretive criteria for *Exiguobacterium* spp., the CLSI criteria for staphylococci were adopted for this study, with *Staphylococcus aureus* ATCC 25923 as the reference strain, in accordance with CLSI document M100 [20]. This approach was chosen because *Exiguobacterium* and staphylococci are both Gram-positive bacteria, permitting the use of similar AST methodologies, and staphylococcal criteria are well-validated and widely accepted in clinical and research settings. However, it should be noted that *Exiguobacterium* and *Staphylococcus* are phylogenetically distant, and intrinsic differences in membrane permeability, efflux pumps, and natural resistance mechanisms may limit the direct applicability of staphylococcal breakpoints.

## 3. Results

### 3.1. Clinical and Pathological Findings in the Deceased Giraffe

**Figure 1:**
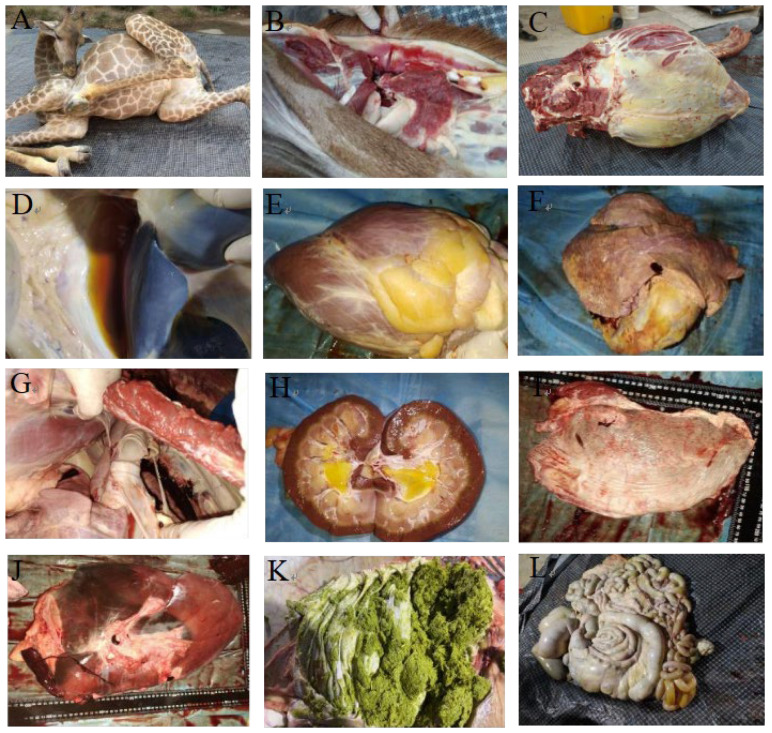
Gross lesions observed in the deceased giraffe. (**A**) Overall view of the giraffe carcass. (**B**) Hemorrhage in cervical lymph nodes. (**C**) Yellow discoloration of subcutaneous muscles. (**D**) Accumulation of fluid in abdominal cavity. (**E**) Yellow gelatinous material on epicardium. (**F**) Marbled appearance of lung surface. (**G**) White froth in tracheobronchial tree. (**H**) Yellow gelatinous material in renal pelvis. (**I**) Pale coloration of spleen. (**J**) Congested liver. (**K**) Greenish gastric contents. (**L**) Yellow intestinal contents.

Clinically, the affected giraffe exhibited marked lethargy and emaciation. Postmortem examination revealed multi-organ pathological changes: hemorrhage in the cervical lymph nodes (Figure 1B); gelatinous subcutaneous edema in the inguinal region and axilla of the right forelimb; and yellow discoloration of the sternal subcutaneous musculature (Figure 1C). Additionally, the ribs were fragile, with a small amount of brownish-yellow effusion in the peritoneal cavity (Figure 1D). The heart (6.5 kg) showed pale myocardium, yellow gelatinous deposits on the epicardium, scattered petechiae, and extensive thrombi in the cardiac chambers (Figure 1E). The lungs (6.5 kg) presented a marbled appearance (Figure 1F), with widened interlobular septa, localized congestion, pale margins, white tracheobronchial froth, and enlarged hilar lymph nodes (Figure 1G). The kidneys (left: 0.8 kg; right: 1.1 kg) displayed uneven coloration, with yellow gelatinous material in the renal pelvis (Figure 1H). The spleen (0.9 kg) was notably pale (Figure 1I), while the liver (6.15 kg) showed mottled discoloration, accompanied by congestion, firm texture, and generalized atrophy (Figure 1J). Other lesions included varying degrees of enlargement of pancreatic lymph nodes; gastric tympany with mucosal sloughing and greenish frothy contents (Figure 1K); abomasal hemorrhage with rice-sized ulcers; orange-yellow fluid in the intestinal lumen (Figure 1L); petechiae in the cecum and colon; and mucoid rectal feces. Collectively, these pathological findings are consistent with a diagnosis of acute bacterial septicemia complicated by multi-organ failure

### 3.2. Histopathological Findings in the Deceased Giraffe

**Figure 2:**
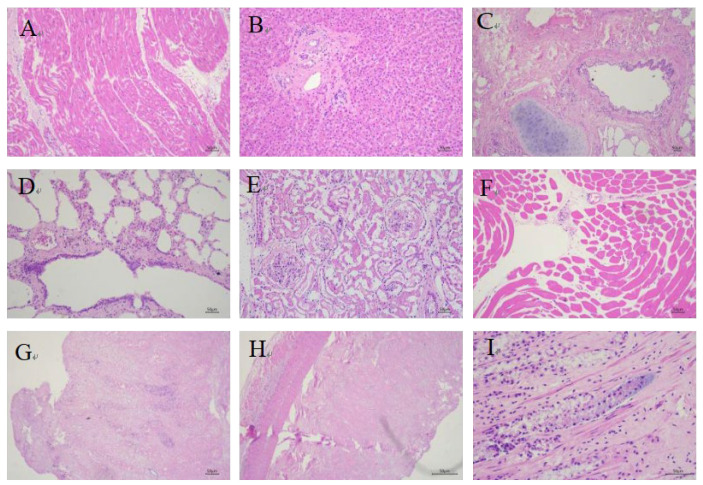
Histopathological lesions in the deceased giraffe (H&E staining). (**A**) Dissolution of myocardial fibers with partial cardiomyocyte degeneration and necrosis. (**B**) Atrophy of hepatic cell cords in the liver. (**C**) Desquamation of bronchial mucosal epithelium with peribronchial lymphocytes and plasma cells. (**D**) Fibrin exudation around blood vessels and presence of macrophages and dust cells in alveoli. (**E**) Degeneration and necrosis of renal tubular epithelial cells with glomerular degeneration and atrophy. (**F**) Skeletal muscle atrophy with disorganized tissue arrangement. (**G**) Transmural necrosis of the gastric wall. (**H**) Transmural intestinal necrosis with nuclear disappearance. (**I**) Dense aggregates of rod-shaped bacteria in intestinal mucosal glands.

Histopathological examination of the deceased giraffe revealed significant pathological changes, characterized by multi-organ atrophy, extensive necrosis, and inflammatory cell infiltration in multiple tissues. Myocardial fibers showed dissolution and disorganization, with partial cardiomyocyte degeneration and necrosis manifested as pyknosis or loss of nuclei (Figure 2A). The liver exhibited atrophied hepatocyte cords with minimal bile pigment deposition, collagen fiber proliferation in the portal triads, and sparse lymphocytic infiltration (Figure 2B). Bronchial mucosal epithelium was desquamated with loss of cilia, accompanied by the peribronchial accumulation of lymphocytes and plasma cells (Figure 2C). Scattered erythrocytes, neutrophils, desquamated epithelial cells, and exudates were observed in pulmonary vessels, along with perivascular fibrin deposition, alveolar macrophages, dust cells, and squamous metaplasia of bronchiolar mucosa (Figure 2D). Renal lesions included degenerative and necrotic tubular epithelial cells with fibrinous casts in the lumens, atrophied glomeruli with capsular degeneration and exudate accumulation, and interstitial inflammatory cell infiltration (Figure 2E). Skeletal muscles displayed atrophy and structural disorganization (Figure 2F). Transmural necrosis was present in the gastric wall, with abundant rod-shaped bacteria observed in the mucosal glands (Figure 2G). Intestinal tissues showed transmural necrosis with disintegration of mucosal epithelium and glandular architecture, blurred cellular boundaries, and nuclear loss (Figure 2H), along with dense aggregates of rod-shaped bacteria in the intestinal glands accompanied by peripheral tissue necrosis (Figure 2I). These histopathological changes indicate severe pyogranulomatous inflammation and extensive tissue necrosis, suggesting that the pathogen is likely an invasive pathogenic bacterium capable of inducing a fatal septic shock-like syndrome.

### 3.3. Bacterium Isolation and Microscopic Examination

**Figure 3:**
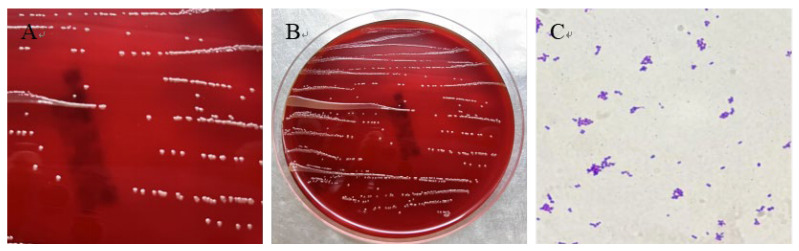
Colonial morphology and microscopic observations of bacterial isolate HN-1. (**A**,**B**) The bacteria grew well on sheep blood agar plates without hemolytic rings. (**C**) Under oil immersion microscopy, Gram-Positive bacilli of varying sizes with inconsistent staining intensity were observed.

Sterilely collected tissue samples were inoculated onto blood agar plate (Biocell, Zhengzhou, China) and LB agar plates for bacterial isolation and culture. A total of 18 bacterial isolates were isolated from heart, spleen, lung, kidney, and liver tissues. One strain, found across multiple tissues, was designated as HN-1. HN-1 colonies were observed to be round, smooth, pale yellow, and raised in morphology (Figure 3A,B). Gram staining identified the newly isolated HN-1 strain as Gram-Positive, displaying a short, rod-shaped morphology with blunt ends, and arranged singly or in pairs (Figure 3C). These characteristics, indicative of systemic infection, suggest that strain HN-1 possesses biological properties consistent with septicemia pathogens.

### 3.4. Strain HN-1 Was Identified as E. mexicanum

**Figure 4:**
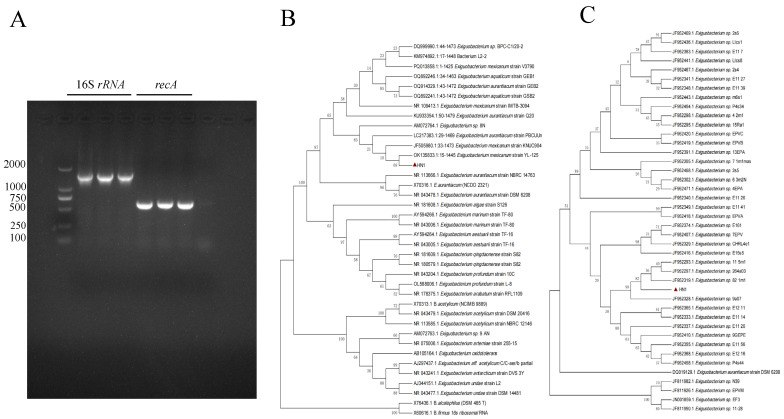
Molecular analysis of HN-1 gene amplicons. (**A**) Agarose gel electrophoresis of 16S *rRNA* and *recA* PCR products from bacterial isolate HN-1. The three lanes for each gene represent amplification products from colonies isolated from three independent culture plates, verifying the reproducibility of the results. (**B**) Phylogenetic tree based on 16S *rRNA* gene sequences. (**C**) Phylogenetic tree based on *recA* gene sequences. Numbers at nodes represent bootstrap support values from 1000 replicate samplings. The triangle represents the isolate in the manuscript.

HN-1 strain underwent further characterization through 16S *rRNA* and *recA* gene sequence analysis. Amplification yielded 16S *rRNA* and *recA* gene fragments of 1446 bp (GenBank accession number: PX248038) and 575 bp (GenBank accession number: PX255259), respectively. The agarose gel electrophoresis of the PCR products (Figure 4A) shows three lanes for both 16S *rRNA* and *recA* gene amplicons, corresponding to bacterial colonies isolated from three independent culture plates, confirming the consistency of the amplification results across different isolates of the same strain. Subsequent BLAST analysis against the NCBI database revealed high sequence similarity between strain HN-1 and various *Exiguobacterium* strains. Specifically, the 16S *rRNA* sequence exhibited 99.73–99.86% similarity, while the *recA* gene sequence showed 98.26–100% similarity. Furthermore, mass spectrometry confirmed that the bacterial strains isolated from different samples were all identified as *E. mexicanum*. Phylogenetic trees, constructed using both 16S *rRNA* and *recA* gene sequences from HN-1 and relevant reference strains, provided further insight into its taxonomic position. The 16S *rRNA* phylogenetic tree (Figure 4B) demonstrated that HN-1 clustered exclusively with *E. mexicanum* OK135833. Likewise, the *recA* phylogenetic tree (Figure 4C) indicated that strain HN-1 formed a distinct clade encompassing all *Exiguobacterium* species included in the analysis. Integrating morphological characteristics with the results of 16S *rRNA* and *recA* gene sequence analyses, as well as confirmation by mass spectrometry, the bacterial strain HN-1 was conclusively identified as *E. mexicanum.*

### 3.5. Antimicrobial Susceptibility Detection

To investigate the antimicrobial susceptibility profile of *E. mexicanum* HN-1, the disk diffusion method was used to determine the susceptibility of this strain to 14 antibiotics. The results showed that strain HN-1 exhibited resistance to oxacillin, penicillin, ciprofloxacin, and linezolid, which was characterized by small inhibition zones. In contrast, the strain was susceptible to levofloxacin, clindamycin, chloramphenicol, trimethoprim, rifampicin, tetracycline, minocycline, gentamicin, erythromycin, and doxycycline (Table 1). These findings not only clarify the resistance characteristics of this strain to certain antibiotics but also provide potential therapeutic options for clinical infections caused by Exiguobacterium mexicanum HN-1.

## 4. Discussion

Global outbreaks of zoonotic diseases have become increasingly frequent in recent decades, posing escalating threats to public health and wildlife conservation [21]. Advances in diagnostic technologies have facilitated the identification of emerging zoonoses—infections caused by bacteria, parasites, viruses, and fungi that originate in animals and are transmitted to humans from wild or domestic reservoirs [18,22]. Within this context, the genus *Exiguobacterium*, a group of Gram-positive bacteria renowned for their exceptional adaptability (including thermotolerance, psychrotolerance, alkaliphily, and halotolerance) [23,24], has garnered increasing attention due to its underrecognized pathogenic potential. Although reports of *Exiguobacterium*-related infections remain scarce, existing evidence underscores their clinical significance. For instance, *E. aurantiacum* has been isolated from blood cultures of bacteremia patients, including those with multiple myeloma, highlighting the heightened susceptibility of immunocompromised individuals [25]. In China, *Exiguobacterium* sp. A1b/GX59 has been associated with community-acquired pneumonia and bacteremia, with the pathogen detected in both blood and bronchoalveolar lavage fluid [11]. These cases align with our findings but also reveal a critical knowledge gap: prior to this study, no cases of *E. mexicanum* infection had been reported in giraffes, nor had systemic infection leading to fatal septicemia been documented. This novelty establishes our work as a pivotal step in expanding the known host range of *E. mexicanum*—previously limited to environmental niches and human clinical cases—to include large ruminants, particularly captive wildlife.

*E. mexicanum*, an opportunistic pathogen ubiquitous in soil and water [26,27], likely infects giraffes through multiple transmission routes such as ingestion of contaminated feed or water, skin wounds, or respiratory exposure [28]. This is consistent with the systemic distribution of strain HN-1 across the heart, liver, spleen, lungs, and kidneys observed in this case. Captive giraffes, already vulnerable to stress-induced immune impairment due to overcrowding or poor sanitation [29], may face elevated risks, as evidenced by the severe pathological lesions—including multi-organ necrosis, thrombus formation, and inflammatory infiltration—identified in our study. Notably, this contrasts with prior research on *E. mexicanum*, which has focused primarily on its role in environmental bioremediation (e.g., nitrogen removal in saline wastewater) [30,31,32] rather than its pathogenicity, underscoring the need to reevaluate its ecological and clinical importance. Currently, antibiotic therapy remains the primary treatment option due to its broad applicability and rapid efficacy [33]. In terms of antimicrobial susceptibility, our findings reveal a distinct profile compared to related species. While six *E. aurantiacum* strains isolated from human blood cultures showed susceptibility to all the tested antibiotics [34], whereas *E. mexicanum* HN-1 showed resistance to oxacillin, penicillin, ciprofloxacin, and linezolid, and susceptibility to levofloxacin, clindamycin, chloramphenicol, trimethoprim, rifampicin, tetracycline, minocycline, gentamicin, erythromycin, and doxycycline. This resistance pattern may reflect local antibiotic usage practices [35] and also emphasizes the importance of conducting species-specific antimicrobial susceptibility testing to avoid ineffective treatments. For the management of captive wildlife, these data provide actionable guidance: prioritizing the use of susceptible antibiotics such as levofloxacin, clindamycin, and gentamicin, while avoiding resistant drugs like penicillin and oxacillin, may improve the cure rate of future cases.

Collectively, this study addresses key gaps in our understanding of *E. mexicanum* pathogenicity. By documenting the first case of *E. mexicanum* infection in giraffes, we confirm that this bacterium can infect large herbivores, thereby expanding its known host range. The observed antibiotic resistance further highlights the need for targeted surveillance in captive settings, where the risk of zoonotic spillover may be amplified. Moving forward, investigations into the bacterium’s virulence factors (e.g., adhesion proteins or toxin production) and the links between environmental reservoirs and animal or human infections will be critical. For zoos and wildlife facilities, our work underscores the urgency of enhancing biosecurity measures—including pathogen monitoring, stress reduction, and prudent antibiotic use—to mitigate risks posed by this emerging pathogen, ultimately safeguarding both animal welfare and public health [36,37,38].

## 5. Conclusions

This study reports on the first case in which *E. mexicanum* was isolated and identified, from a deceased giraffe, with strain HN-1 exhibiting resistance to penicillin, oxacillin, ciprofloxacin, and linezolid while being susceptible to most other tested antibiotics. The systemic distribution of HN-1 in multiple visceral organs and the severe pathological changes observed suggest its potential role in the giraffe’s fatal septicemia. However, it is important to acknowledge that 18 bacterial strains were isolated from the animal, and only HN-1 was characterized. Thus, we cannot rule out the possibility of a mixed infection, where other unstudied strains may have contributed to the pathogenesis. Future research should focus on the comprehensive identification of all isolates, assessment of their pathogenicity in combination, and exploration of virulence factors to clarify the exact etiology of such infections in giraffes. These findings provide a foundation for understanding *E. mexicanum* infections in large ruminants and offer guidance for clinical treatment in captive wildlife settings.

## Figures and Tables

**Table 1:** Antimicrobial sensitivity analysis results of isolated strain.

Antibiotics	Medication Dose (ug/disk)	IZD (mm)	Sensitivity	Judgment Standard of Inhibition Zone Diameter (mm)		
				Resistant	Intermediate	Sensitive
Gentamicin	10	21.1	S	≤12	13~14	≥15
Ciprofloxacin	5	12.4	R	≤15	16~20	≥21
Chloramphenicol	30	24	S	≤12	13~17	≥18
Erythromycin	15	20.5	I	≤13	14~22	≥23
Doxycycline	30	21	S	≤12	13~15	≥16
Levofloxacin	5	20	S	≤15	16~18	≥19
Tetracycline	30	26	S	≤14	15~18	≥19
Minocycline	30	27	S	≤14	15~18	≥19
Trimethoprim	5	10.5	I	≤10	11~15	≥16
Clindamycin	2	22	S	≤14	15~20	≥21
Linezolid	30	15.2	R	≤22	23~25	≥26
Rifampicin	5	17	I	≤16	17~19	≥20
Oxacillin	1	9.7	R	≤24	—	≥25
Penicillin	10	16.6	R	≤28	—	≥29

Note: S: susceptible, I: intermediate, R: resistant.

## Data Availability

The original contributions presented in this study are included in the article. Further inquiries can be directed to the corresponding authors.
